# Resolution of immune thrombocytopenic purpura associated with extranodal B-cell lymphoma of the petroclival region after radiotherapy

**DOI:** 10.4103/2152-7806.73318

**Published:** 2010-11-27

**Authors:** Takashi Watanabe, Hideyuki Kurihara, Satoshi Magarisawa, Shigeru Shimoda, Katsue Yoshida, Shogo Ishiuchi

**Affiliations:** Department of Neurosurgery, Faculty of Clinical Medicine, University of the Ryukyus, Nakagami-gun, Okinawa, Japan; 1 Department of Neurosurgery and Pathology, Kiryu Kousei Hospital, Kiryu, Gunma, Japan

**Keywords:** B-cell lymphoma, immune thrombocytopenic purpura, radiotherapy, skull base

## Abstract

**Background:**

Secondary immune thrombocytopenic purpura (ITP) associated with extranodal B-cell non-Hodgkin’s lymphoma (NHL) is extremely rare. The optimal management is not established. We report a first case of ITP in association with extranodal B-cell NHL originating in the lower petroclival region, successfully managed by local tumor control using conventional radiotherapy.

**Case Description:**

A 75-year-old man presented with a two-month history of hearing loss, hoarseness, and dysphagia. Neuroimaging revealed a large enhanced lesion in the left lower petroclival bone near the jugular foramen. Isolated unilateral parotid lymphadenopathy was also noted. Preoperative laboratory findings were normal, except for elevation of serum soluble interleukin-2 receptor level. A suboccipital craniotomy was performed and a biopsy sample was taken. Histological and immunohistochemical examination confirmed small B-cell lymphoma with plasmacytic differentiation. After initiation of radiotherapy, thrombocytopenia (24,000/µl) rapidly developed. Serological and bone marrow examination confirmed ITP. Prednisone was given at 1 mg/kg/day and radiation therapy was continued. After more than 32Gy, platelet count rapidly normalized. Radiotherapy to the tumor site achieved local tumor control and ITP was resolved. No evidence of recurrence and normal platelet count were confirmed at the two-year follow-up examination.

**Conclusion:**

Local control of the tumor was considered important in the resolution of secondary ITP in association with extranodal NHL of the skull base region.

## INTRODUCTION

Immune thrombocytopenic purpura (ITP) is a type of autoimmune thrombocytopenia associated with antibody-mediated accelerated platelet destruction, characterized by low platelet count and hemorrhagic tendency in the skin or submucosa despite normal or overactive platelet production.[2] In primary ITP, the underlying diseases or causes are not detected. Secondary ITP occurs in association with systemic lupus erythematosus, antiphospholipid syndrome, immunodeficiency status, and lymphoproliferative disorders, including lymphoma.[1,2,10,12] ITP associated with B-cell non-Hodgkin’s lymphoma (NHL) is rare, with only 33 reported cases,[6] so the prognosis and optimal management are poorly understood.

We describe a first case of secondary ITP associated with lower petroclival B-cell NHL, which was successfully managed by local tumor control using conventional radiotherapy.

## CASE REPORT

**Figure 1:**
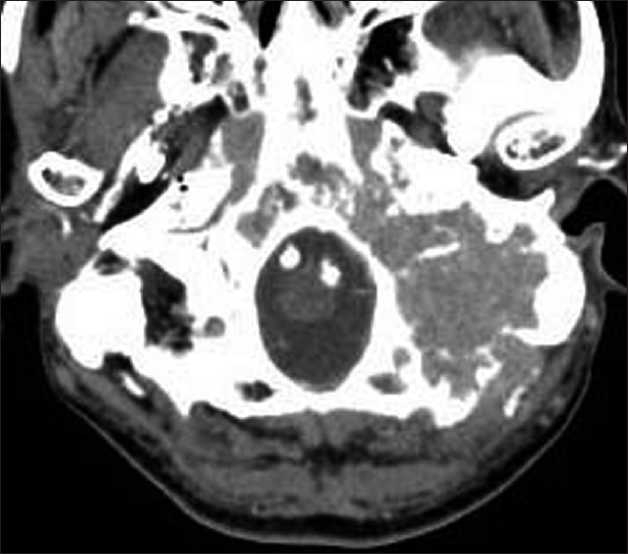
CT scan with contrast medium of the head showing a homogeneously enhanced lesion in the clivus and petrous bone with extensive osteolytic reaction

**Figure 2:**
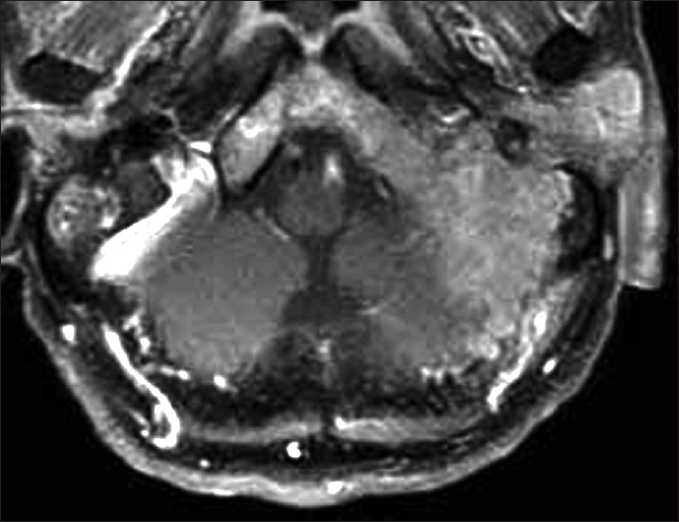
Gadolinium-enhaced T1-weighted MR image of the brain demonstrating an enhancing mass with diffuse infiltration into the clivus and petrous bone

A 75-year-old male presented with loss of hearing, hoarseness, and dysphagia, progressively deteriorating over two months. The patient had a past history of diabetes mellitus and hypertension. Clinical examination found a painless left parotid lymphadenopathy and a paralysis of the soft palate and tongue on the affected side. Audiography showed Class 2 hearing on the Gardner–Robertson scale[4] in the affected ear. No other abnormalities were identified. Blood cell counts and serological examination were normal, except for elevation of soluble interleukin-2 receptor level at 2950 U/ml (normal range 220–530 U/ml). Computed tomography (CT) with contrast medium revealed a slightly enhanced mass, maximum diameter 6 cm, in the clivus and petrous bone with extensive osteolytic reaction [Figure 1]. Magnetic resonance imaging (MRI) demonstrated a tumor diffusely infiltrating into the clivus and petrous bone with homogeneous enhancement after gadolinium administration [Figure 2]. Both CT and MRI revealed isolated unilateral parotid lymphadenopathy. Cerebral angiography demonstrated tumor staining supplied by the ascending pharyngeal artery and occipital artery. Positron emission tomography using fluorine-18 fluoro-2-deoxyglucose revealed strong uptake in the clivus. Gallium-67 scintigraphy and contrast-enhanced CT scan of the chest, abdomen, and pelvis confirmed petroclival origin.

**Figure 3:**
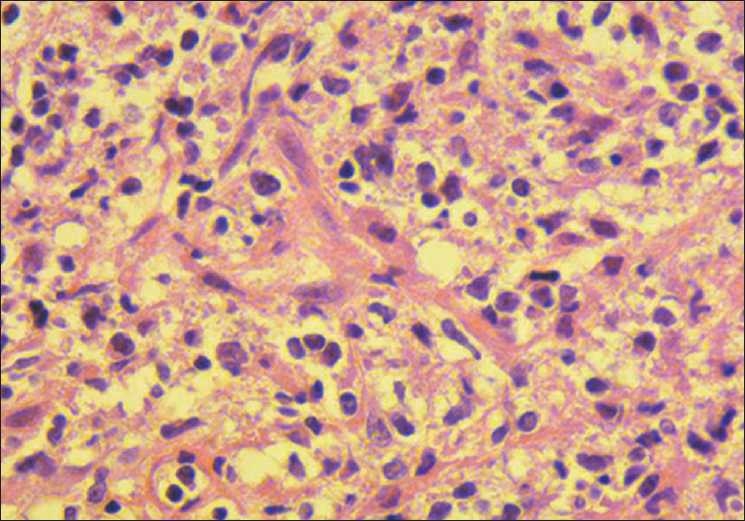
Photomicrograph demonstrating lymphoma cells consisting of small to medium lymphocytes and plasmacytoid cells with abundant basophilic cytoplasm and lymphocyte-like nuclei. (H & E, ×400)

A suboccipital craniotomy was performed and a biopsy sample was taken. The tumor had infiltrated into the suboccipital muscles and eroded through the occipital bone and petrous bone. The dura mater was not involved. Histological analysis of the biopsy samples demonstrated the diffusely infiltrated tumor in the muscles and bone, which consisted of small to medium lymphocytes and plasmacytoid cells with abundant basophilic cytoplasm and lymphocyte-like nuclei [Figure 3]. Immunohistochemical examination demonstrated the tumor cells were immunopositive for B-cell-associated antigens LCA, CD20, and CD79a, and immunonegative for UCHL-1, CD3, CD5, CD10, and CD23. Based on these findings, the diagnosis was small B-cell lymphoma with plasmacytic differentiation.

**Figure 4:**
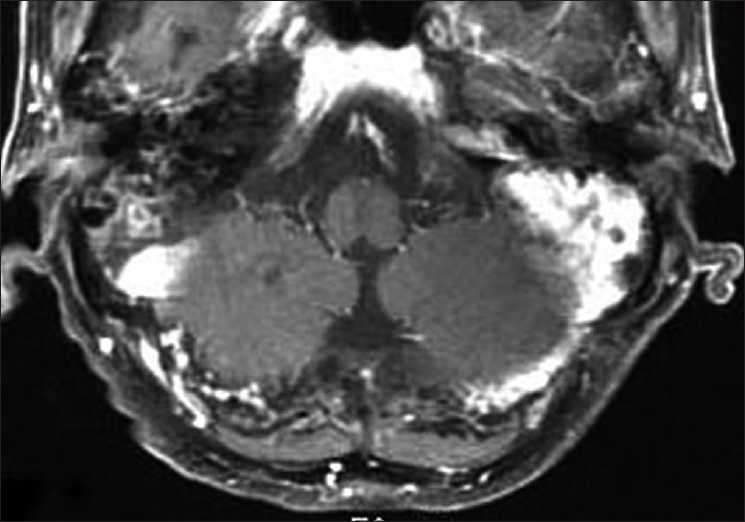
MR image after radiotherapy revealing complete remission

The postoperative course was uneventful. Laboratory examination just after surgery found no abnormalities. Local radiation therapy at 2Gy/fraction was started one week after surgery. Parotid lesion was also included in the field of radiation. Complete blood count after 14Gy irradiation revealed thrombocytopenia with platelet count of 38,000/*µ*l, hemoglobin of 11.7 g/dl, and white blood cell count of 5200/*µ*l. On the next day, platelet count fell to 24,000/*µ*l. Serum biochemistry and serum protein electrophoresis revealed findings within normaL limits. Serum M-component was not detected. Bence–Jones protein was not found in the urine. Platelet-associated immunoglobulin G was 254 ng/10^7^cells (normal range 9–25 ng/10^7^cells). Other autoantibodies including anticardiolipin and lupus anticoagulant were not detected. Serological tests for recent viral infection (hepatitis B virus, hepatitis C virus) were negative. Results of bone marrow aspiration showed normal cellularity and increase in megakaryocytes. Drug-induced thrombocytopenia was also excluded. The diagnosis was immune thrombocytic purpura. Oral prednisone 1 mg/kg/day was initiated and was gradually tapered within a few weeks. Platelet count rose to 90,000/*µ*l and 220,000/*µ*l after 32Gy and 46Gy irradiation, respectively. Finally, a total dose of 50Gy was delivered to the entire lesion, and a booster dose of 6Gy was added to the residual lesion in the clivus. Hoarseness and dysphagia improved to allow eating of normal food. Magnetic resonance imaging after radiotherapy confirmed complete remission [Figure 4]. No evidence of recurrence and normal platelet count were confirmed at the two-year follow-up examination.

## DISCUSSION

Acquired ITP has developed in association with various NHL subtypes, including small lymphocytic lymphoma, follicular lymphoma, marginal cell lymphoma, mantle cell lymphoma, hairy cell lymphoma, lymphoplasmacytic lymphoma, and high grade B-cell lymphoma.[6] Immune thrombocytopenic purpura occurring after the diagnosis of NHL is refractory to oral prednisone, immune suppressive therapy, and splenectomy, and may be life threatening.[12] The appropriate management is not established. Review of the 33 cases with ITP associated with NHL found no sustained complete remission after only steroid therapy, but complete remission was achieved in more than half of the patients after anti-lymphoma treatment, such as removal of the lymphoma or anti-lymphoma chemotherapy.[6] One case of ITP regressed five days after endoscopic mucosal resection of a gastric mucosa-associated lymphoid tissue (MALT) lymphoma without *Helicobacter pylori* eradication.[10] Another case of relapsing refractory ITP associated with low grade NHL was successfully treated with the monoclonal anti-CD20 antibody, rituximab.[12] These findings suggest that anti-lymphoma treatment may be effective for acquired ITP associated with NHL.

Malignant lymphoma may involve extranodal sites of the head and neck region, and arise most commonly in association with Waldeyer’s ring and ocular adnexal structures.[14] Extranodal NHL originating in the clivus is extremely rare.[3,5,7,13] The present case of ITP in association with extranodal NHL occurring in the petroclival region is thus extremely rare. The treatment of extranodal lymphoma of head and neck region depends on the histological subtype and primary site of organ involvement. Patients with early-stage extranodal indolent NHL are good candidates for radiation therapy. Radiation doses of 30–40Gy are considered adequate to eradicate the lesion.[9,11] The in-field failure rate increases progressively with tumor size, suggesting that larger tumors (>6 cm) may need to be treated with higher radiation doses.[8] The present patient with stage II_E_ indolent B-cell lymphoma with tumor size of 6 cm was treated with radiation therapy using a total dose of 50Gy to the involved field followed by 6Gy boost to the residual lesion in the clivus. Complete hematological remission was achieved after 32Gy irradiation, which is thought to be high enough to control the tumor. No evidence of relapse and normal hematological findings within normal limits were found at the two-year follow-up examination despite tapering of the prednisone therapy. These findings suggest that local tumor control is important in remission of ITP associated with localized indolent NHL. Radiation therapy should be continued until local tumor control is achieved, despite rapidly developing thrombocytopenia in patients with extranodal NHL. Close follow-up examinations are necessary for early detection of recurrence. Although the long-term follow-up result and effectiveness of other treatment modalities are unclear, chemotherapy using anti-CD20 antibody, rituximab may be a treatment of choice for recurrence.

## CONCLUSION

The present case of ITP associated with extranodal B-cell lymphoma originating in the lower petroclival lesion was resolved after local tumor control achieved by conventional radiotherapy. Local tumor control may be important in treating ITP associated with localized indolent extranodal NHL.
